# Metabolomics of Early Stage Plant Cell–Microbe Interaction Using Stable Isotope Labeling

**DOI:** 10.3389/fpls.2018.00760

**Published:** 2018-06-05

**Authors:** Qiuying Pang, Tong Zhang, Yang Wang, Wenwen Kong, Qijie Guan, Xiufeng Yan, Sixue Chen

**Affiliations:** ^1^Key Laboratory of Saline-alkali Vegetation Ecology Restoration in Oil Field, Ministry of Education, Alkali Soil Natural Environmental Science Center, Northeast Forestry University, Harbin, China; ^2^Department of Biology, Genetics Institute, University of Florida, Gainesville, FL, United States; ^3^Plant Molecular and Cellular Biology Program, University of Florida, Gainesville, FL, United States; ^4^Proteomics and Mass Spectrometry, Interdisciplinary Center for Biotechnology Research (ICBR), University of Florida, Gainesville, FL, United States

**Keywords:** plant–microbe interaction, stable isotope labeling, metabolomics, *Pst* DC3000, stomatal defense

## Abstract

Metabolomics has been used in unraveling metabolites that play essential roles in plant–microbe (including pathogen) interactions. However, the problem of profiling a plant metabolome with potential contaminating metabolites from the coexisting microbes has been largely ignored. To address this problem, we implemented an effective stable isotope labeling approach, where the metabolome of a plant bacterial pathogen *Pseudomonas syringae* pv. tomato (*Pst*) DC3000 was labeled with heavy isotopes. The labeled bacterial cells were incubated with *Arabidopsis thaliana* epidermal peels (EPs) with guard cells, and excessive bacterial cells were subsequently removed from the plant tissues by washing. The plant metabolites were characterized by liquid chromatography mass spectrometry using multiple reactions monitoring, which can differentiate plant and bacterial metabolites. Targeted metabolomic analysis suggested that *Pst* DC3000 infection may modulate stomatal movement by reprograming plant signaling and primary metabolic pathways. This proof-of-concept study demonstrates the utility of this strategy in differentiation of the plant and microbe metabolomes, and it has broad applications in studying metabolic interactions between microbes and other organisms.

## Introduction

Plant-microbe interactions involve a series of exchange of chemicals for signal perception, transduction, and metabolic responses. During pathogen infection, plant cells detect pathogen-associated molecular patterns (PAMPs), which lead to the production of specialized metabolites such as phytoalexins to combat the pathogen invasion (Lin et al., 2014; Poloni and Schirawski, 2014; Arbona and Gomez-Cadenas, 2016). Past studies have also demonstrated that reprograming of the primary metabolic pathways contributes to the plant defense against pathogens. For example, modulation of photosynthesis and other primary plant metabolic pathways such as amino acid and lipid metabolism has been associated with altered plant immune responses (Berger et al., 2007; Bolton, 2009; Rojas et al., 2014). Thus, the regulation in primary metabolism such as photosynthesis, assimilate partition and source-sink regulation, as well as the production of specialized metabolites in plant–pathogen interactions has become an emerging research topic.

To systematically analyze plant immunity-related metabolites, metabolomics has shown utility due to its ability to identify and quantify hundreds of compounds simultaneously (Misra et al., 2016; Lima et al., 2017). However, a major problem with current metabolomics approaches in studying plant–pathogen interactions is the difficulty to discern plant metabolites from the pathogen metabolites. In a typical assay, plant materials are incubated with pathogens, which can attach to and/or get into the plant tissues. Current studies usually do not differentiate microbial metabolites from plant metabolites since there was little or no effort to remove the microbes before metabolite extraction (Camañes et al., 2015; Qian et al., 2015; Suharti et al., 2016; Lima et al., 2017). While this is not a problem for transcriptomics and proteomics when species specific databases are available, cross-contamination between the plant metabolome and the microbial metabolome is a serious issue. In spite of limited attempts to quickly separate the bacterial cells from the infected plants, it is impossible to completely remove bacterial cells (Allwood et al., 2010, 2012). The presence of a broad range of shared metabolites such as carbohydrates, amino acids and nucleic acids adds another layer of complexity to quantify metabolic changes in either interacting partners.

Culturing cells in stable isotope media is a powerful way to trace the origin of biomolecules. For example, stable isotope labeling by amino acids in cell culture (SILAC) has been utilized in labeling the proteomes of bacterial cells (Soufi et al., 2010; Soufi and Macek, 2014). Similarly, stable isotope labeling can be applied for reference metabolite labeling (e.g., for accurate quantification), metabolic flux analysis and identification of metabolites in different organisms (Creek et al., 2012; Bueschl et al., 2013; Chokkathukalam et al., 2014; You et al., 2014; Silva et al., 2016). For example, 13C-labeled hexanoic acid was applied to citrus plants to track the emission of plant volatiles and avoid interference from the endogenous compound (Llorens et al., 2016). However, isotope labeling has not been applied to studying plant-microbe interactions. Our lab is interested in the signaling and metabolic processes underlying plant innate immunity using *Arabidopsis thaliana* epidermal peels (EPs) and *Pseudomonas syringae* pv. tomato (*Pst*) DC3000, a model system for studying plant pathogen interactions (Xin and He, 2013). For *Pst* DC3000 to cause infection, they need initial contact with epidermis and enter through stomatal pores formed by pairs of guard cells. When guard cells sense the bacterial PAMPs, they quickly close the stomata within 1 h as an innate immunity response. However, many bacterial pathogens such as *Pst* DC3000 can re-open stomata in 3 h to facilitate entry into plants through secretion of coronatine (COR) (Melotto et al., 2006; Zhang et al., 2008; Arnaud and Hwang, 2015; Panchal et al., 2016). COR secretion is a good indication of the interaction between *Pst* DC3000 and plants (Melotto et al., 2006). Since COR is structurally similar to jasmonic acid isoleucine (JA-Ile), COR was thought to antagonize and dampen the salicylic acid (SA) mediated defense (Zheng et al., 2012).

To analyze species-specific metabolites during the early stage of plant–pathogen interaction, we report a strategy that combines metabolic labeling of *Pst* DC3000 with stable isotopes and rapid reduction of the bacterial cells from the Arabidopsis EPs through salt washing. Allwood et al. (2010, 2012) showed that after bacterial incubation with plant suspension cells, salt washing was very efficient in reducing the number of bacteria cells associated with the plant cells. Here we show that quick washing with salt is indeed an efficient way to remove most *Pst* cells after incubation with the EPs. We also demonstrate that isotopic labeling provides an effective way to distinguish plant metabolites from the *Pst* metabolites.

## Materials and Methods

### Plant Materials

Arabidopsis Col-0 seeds were obtained from the Arabidopsis Biological Resource Center, and were germinated on a half-strength Murashige and Skoog (1962) medium prior to transferring the young seedlings to a Metro-Mix MVP soil (The Scotts Co., Marysville, OH, United States) in a Percival growth chamber (Percival Scientific Inc., Perry, IA, United States). Plants were grown under a photosynthetic flux of 140 μmol photons m^-2^ sec^-1^ and an 8 h light/16 h dark cycle for 5 weeks. EPs with enriched stomatal guard cells were prepared as described previously (Zhang et al., 2016; Zhu et al., 2017). Briefly, 30 g of leaves from ∼50 plants (three leaves/plant, one leaf weighs approximately 0.2 g) were blended in tape water for 30 s, and the EPs were collected by filtering the mixture through a mesh (100 μm in pore size). Pavement cells were selectively digested in an enzyme cocktail containing 0.7 % Cellulase R-10 and 0.025% Macerozyme R-10 (Yakult Honsha Co., Ltd., Tokyo, Japan). The enriched guard cell samples were drained and blotted dry briefly. For each replicate, 100 mg of EPs (with primarily stomatal guard cells) were incubated in 10 mM KCl, 50 μM CaCl_2_, 10 mM MES-KOH, pH 6.15 under light (140 μmol photons m^-2^ sec^-1^) for 3 h. Three biological replicates were conducted for all experiments in this study.

### Bacterial Culture and Isotopic Labeling

*Pst* DC3000 was cultured at 28°C in a Luria-Bertani (LB) medium (5 g/L of yeast extract, 10 g/L of tryptone, and 10 g/L of NaCl) supplemented with 25 mg/L rifampicin and 50 mg/L kanamycin until an OD_600_ of 0.8 was reached. For isotope labeling, a single colony was inoculated to 1 ml of an isotope-labeled medium (Celtone complete medium with ^13^C, 98%+ and ^15^N, 98%+) (Cambridge Isotope Laboratories, Tewksbury, MA, United States) and shaken at 220 rpm overnight. Two successive subcultures were made by inoculating fresh heavy isotope medium with the previous generation of culture at 1:100 ratios (bacteria: media). Control experiments were done in the same way except that regular LB medium was used. The resulting bacteria were collected by centrifugation at 4000 rpm for 1 min, and the pellets were reconstituted in water to a final concentration of 10^8^ colony forming unit (cfu)/ml.

### Plant–Pathogen Interaction, Separation and Sample Collection

The plant pathogen interaction assays were performed by incubating 100 mg of EPs with 2 mL *Pst* DC3000 (10^8^/ml water) for different lengths of time. For mock controls, water was used to incubate with the EPs. To remove bacterial cells after incubation at indicated time points, washing was conducted to *Pst* DC3000 treated EPs and mock EPs with 0.85% NaCl (w/v) as described (Allwood et al., 2010, 2012) with minor modifications. Briefly, the mixture was filtered through a 100 μm nylon mesh using a Buchner funnel. Vacuum was applied to facilitate liquid removal from the EPs. The samples were blot-dry briefly after washing and then transferred to 1.5 mL tubes. To assess the washing efficiency, the samples were ground in 500 μl 10 mM MgCl_2_ with a plastic pestle, and the resulting mixture was diluted 10,000 times before plating onto a King’s B medium (20 g/L of peptone, 1.5 g/L of K_2_HPO_4_, 0.75 g/L MgSO_4_, 10 mL/L glycerol and 15 g/L agar) for cfu analysis. The washed samples were frozen in liquid nitrogen and stored in -80°C before metabolite extraction.

### Metabolite Profiling

Metabolite extraction and liquid chromatography-multiple reaction monitoring-mass spectrometry (LC-MRM-MS) were conducted as previously described (Misra et al., 2015, 2016; Geng et al., 2016). Briefly, samples from three biological replicates were analyzed on an Agilent 1100 HPLC (Agilent, Santa Clara, CA, United States) coupled with an AB Sciex 4000 QTRAP^TM^ (AB Sciex, Framingham, MA, United States). A reverse-phase C18 column (Agilent, Eclipse XDBC18, 4.6 × 250 mm, 5 μm) was used for metabolite separation with 0.1% formic acid in water as solvent A and 0.1% formic acid in acetonitrile as solvent B. The LC gradient was initially held at 1% of B for 5 min, then a linear gradient was imposed from 1 to 99.5% of B over 41.5 min, followed by holding at 99.5% of B for 4.5 min, and then return to 1% of B. The flow rate was 0.5 ml/min, and the total run time was 1 h. The MS conditions were: 30 psi for curtain gas, 50 psi GS1, 55 psi GS2, ion source voltage at ±4500 V, with the turbo electro spray ionization (ESI) interface temperature at 350°C. Parameters including declustering potential, collision energy and cell exit potential for the MRM transitions were described previously (Geng et al., 2016). Quantification of the metabolites was performed with MultiQuant 2.1 (AB Sciex Inc., Foster City, CA, United States).

To evaluate the isotope incorporation in the *Pst* DC3000 culture, bacterial cells were cultured in the heavy medium only. After metabolite extraction, selected compounds were quantified by LC-MRM-MS with both light and heavy metabolite transitions included in the list. The labeling efficiency was calculated as peak areas of the heavy metabolites divided by the sum of corresponding peak areas of the light metabolites and the heavy metabolites. For COR quantification, MRM transitions were optimized using authentic standards from Sigma-Aldrich (St. Louis, MO, United States) and the LC-MS described above with both light and heavy COR transitions. To induce COR secretion, Light or heavy *Pst* DC3000 was incubated with EP samples for 0, 30, 60, 120, and 180 min. Extraction of COR in the incubation media was performed according to a previous method (Palmer and Bender, 1993). The COR concentrations were calculated based on the COR standard curve (**Supplementary Figure 1**).

### Data Analysis

The quantification data from MultiQuant were exported to Microsoft Excel as csv files and statistical analysis was performed using R (version 3.3.3). Metabolites that have missing values (not identified) in the samples were removed from further analysis. The peak areas of lidocaine and 10-camphorsulfonic acid were used for normalization of metabolites identified in the positive and negative mode, respectively. The reproducibility of the LC-MS system was also evaluated by calculating the coefficient of variation (cv) of the standard compounds from different samples. A heatmap to show the relative metabolite abundance in bacterial and plant samples was generated using the heatmap.2 function in the “gplots” package. The raw abundance was log10 transformed to improve comparison of metabolites with high and low intensity, and the hierarchical clustering analysis was presented along with the heat map. Principle component analysis (PCA) and volcano plot analysis were performed using the R base package. Pathway analysis was conducted using MetaboAnalyst 3.0 (Xia et al., 2015) with *A. thaliana* as the reference pathway library. Significance of the pathway enrichment was tested using Fisher’s exact method.

## Results

### Interaction Between *Pst* DC3000 and *A. thaliana* EPs

**FIGURE 1:**
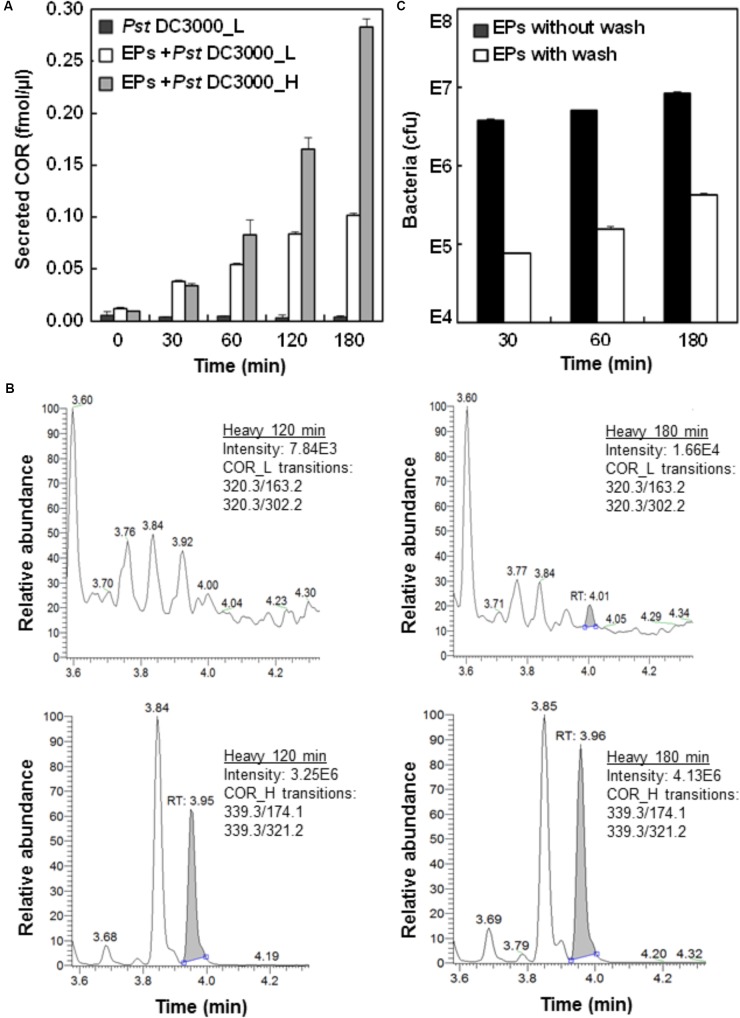
Analysis of *Pst* DC3000 interaction with Arabidopsis epidermal peels (EPs). **(A)** *Pst* DC3000 induces the secretion of COR. Arabidopsis EPs were incubated with light (L) and heavy (H) isotope-labeled *Pst* DC3000, and the secretion of COR_L and COR_H into the media was measured by liquid chromatography-multiple reaction monitoring-mass spectrometry (LC-MRM-MS). The peak abundance data were used to deduce the concentration based on standard curves with authentic compounds (**Supplementary Figure 1**). **(B)** MRM chromatograms of COR_L (top) and COR_H (bottom) secreted by the *Pst* DC3000_H after incubation with Arabidopsis EPs in water for 120 and 180 min. **(C)** Separation of *Pst* DC3000 from plant materials by salt washing. Arabidopsis EPs were washed with 0.85% NaCl extensively after incubation with *Pst* DC3000 for indicated time periods. Samples before and after washing were subjected to bacterial growth assay, and the colony forming unit (cfu) data were presented based on three biological replicates. Standard errors from three replicates were calculated and plotted as error bars in **A,C**.

To ascertain the biological relevance of the *Pst* DC3000-EPs interaction, we first incubated *Pst* DC3000 with EPs of stomatal guard cells and monitored the production of COR. As a mimic of the plant hormone JA-Ile, COR functions through activating the JA signaling pathway, thus antagonizing and dampening the SA-mediated plant defense (Melotto et al., 2006; Panchal et al., 2016). COR secretion by *Pst* DC3000 accumulated to a significantly high level at 1 h after incubation (**Figure 1A**), which is in line with previous observations that COR reopens the PAMP-triggered stomatal closure (Melotto et al., 2006; Panchal et al., 2016). To test heavy COR secretion, heavy labeled *Pst* DC3000 were incubated with EPs for a total of 3 h. The secretion of heavy COR followed the same trend as the light COR, except at a much higher levels at the later time points (**Figure 1A**). Interestingly, over the 3 h incubation period in water, only trace amount of light coronatine (about 1:3000 of heavy) could be detected (**Figure 1A**), confirming the durability of the heavy label and indicating very low reproduction rate of the bacteria on the peels in water.

To determine the number of bacterial cells associated with the EPs after different periods of incubation, a bacterial growth assay was used (Melotto et al., 2006). The results showed that close to 10^7^ bacteria coexist with each plant sample (**Figure 1C**), indicating the close physical association of *Pst* DC3000 with the EPs during the interaction. To remove *Pst* DC3000 for EP metabolite profiling, we adapted a previous method using salt washing (Allwood et al., 2012). As shown in **Figure 1B**, at least 95% of bacterial cells can be removed after three quick washes with 0.85% NaCl. However, the remaining *Pst* DC3000 still represented about 10^5^ bacterial cells per 100 mg EPs, which was significantly different from a few hundred cells per sample (around 100 mg) as reported before (Allwood et al., 2012). One possible explanation is that *Pst* DC3000 can be trapped easily in the apoplast and stomatal pores of EPs, which is not the case for the suspension cell cultures used by Allwood et al. (2012). Nevertheless, our EP system represents a real plant cell type, i.e., stomatal guard cells.

### Metabolic Profiling of *A. thaliana* EPs-*Pst* DC3000 Interaction

**FIGURE 2:**
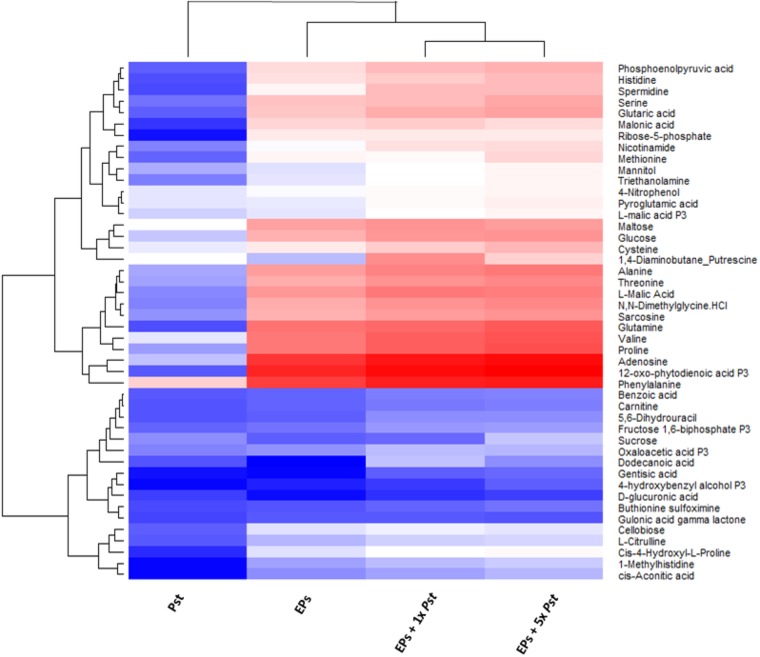
Heat map analysis of the relative abundances of common metabolites between *Pst* DC3000 and Arabidopsis. The levels obtained from MaxQuant were normalized and then log10 transformed, and quantification data of metabolites identified in all the samples were presented. Blue and red denote low and high abundance, respectively. The bottom cluster contains metabolites that have similar levels in plants and bacteria.

To determine the contribution of the remaining 10^5^ bacterial cells to the plant metabolome, we empirically analyzed the metabolite profiles of *Pst* DC3000, and EPs with and without *Pst* DC3000, respectively. We used an established MRM-based targeted metabolomics approach for the analyses (Misra et al., 2015, 2016; Geng et al., 2016). Stringent analysis was performed to prevent false positive identification of metabolites and the results showed that 57 metabolites can be identified confidently from 10^5^ bacteria (**Supplementary Table 1**). These metabolites were also detected from the EPs, and EPs with *Pst* DC3000. When the peak areas of these metabolites were log transformed and plotted on a heat map, it was clear that the abundance of most of them are higher in the EP and EP with 10^5^ *Pst* DC3000 samples (**Figure 2**). An EP sample with 5 × 10^5^ *Pst* was included in this analysis to reveal the significance of the contribution from *Pst* DC3000. Notably, The heatmap also revealed that many shared metabolites (e.g., sugars and amino acids) showed comparable levels in plants and bacterial samples. Thus, there is an urgent need to separate bacterial metabolites to truly reveal the changes of plant metabolites in response to the pathogen invasion.

### Development of Isotope-Labeling of *Pst* DC3000 to Discern Species-Specific Metabolites

**FIGURE 3:**
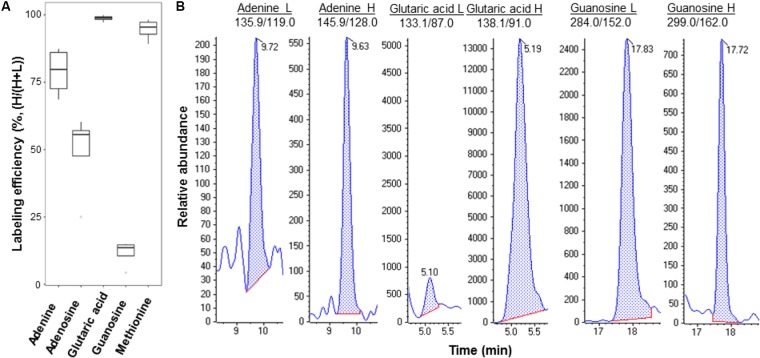
Example metabolites quantified from *Pst* DC3000 cultured in isotope-labeled medium. **(A)** Labeling efficiency profiles of five *Pst* DC3000 metabolites. The distribution of heavy labeled percentage [H/(H+L)] was plotted in the boxplot, where the upper and lower boundary of the box represented the first and third quantile, respectively, of the experimental data. **(B)** Examples of MRM chromatograms of light and heavy labeled adenine, glutaric acid, and guanosine from the heavy isotope-labeled *Pst* DC3000.

We culutred the *Pst* DC3000 with the heavy isotope medium shown to be effective for isotope labeling (Haugstetter et al., 2007; Amniai et al., 2011; Acedo et al., 2015). Incorporation of both ^13^C and ^15^N results in metabolite mass shift, but still maintains the retention on reverse phase LC columns. After establishing the MRM transitions for the heavy and light metabolites (**Supplementary Table 2**), the light and heavy metabolite pairs can be profiled in a single LC-MS run. We first evulated the labeling efficiency of *Pst* DC3000 by quantifying the light and heavy metabolites in the bacteria cultured in the heavy isotope medium. If the labeling efficiency is 100%, the peak areas of the heavy metabolites divided by the sum of corresponding peak areas of the light metabolites and the heavy metabolites should reach to 1, i.e., no light metabolites can be detected. As expected, the results showed that the selected metabolites can be identifed from the isotope-labeled samples, and different metabolites exhibited different labeling efficiencies (**Figure 3**), ranging from a few percent to almost 100% (**Figure 3B** and **Supplementary Figure 2**). This may be due to unlabeled CO_2_ entering the system to make labeling vary from compound to compound. Although 100% labeling was not achieved, we can determine the amount of bacterial metabolites by correcting for the labeling variation.

### Changes of the EP Guard Cell Metabolites in Response to *Pst* DC3000

**FIGURE 4:**
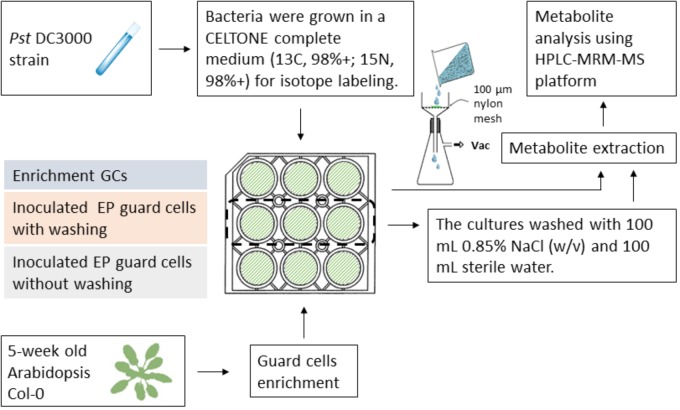
Workflow of profiling plant-derived metabolites in studying plant–pathogen interaction. *Pst* DC3000 cells were cultured in a Celtone complete medium for heavy isotope incorporation. The resulting bacteria were incubated with Arabidopsis EPs. Vacuum-assisted filtration was used to remove *Pst* DC3000 from EPs after indicated incubation time, and the remaining EPs were subjected to metabolite analysis.

Since isotope labeling of *Pst* DC3000 allows determination of metabolites of different origins during the plant–pathogen interactions and the vacuum-facilitated salt washing is effective in reducing the bacterial pathogen, we developed a workflow to profile the plant-specific metabolomic changes. As shown in **Figure 4**, several key steps were integrated: (i) culturing the infecting pathogens in the stable isotope media, (ii) removing most of the bacterial cells by vacuum-facilitated salt washing, and (iii) profiling metabolites using MRM transitions for non-isotope-labeled compounds (for plant metabolites only). Heavy-isotope-labeled compound from the bacteria would not be detected using the transitions for non-labeled metabolites (Geng et al., 2016).

Following this workflow, we incubated *Pst* DC3000 with *A. thaliana* EPs and aimed to characterize the pathogen induced metabolic changes in the EP guard cells. The raw intensity data in **Supplementary Data 1** was first normalized according to the internal standard compounds lidocaine and 10-camphorsulfonic acid, which showed an average cv of 2.9% and 0.35%, respectively. Reproducibility among replicates was assayed by Pearson’s correlation with *R*^2^ ranged from 0.94 and 0.98, demonstrating high quantification repeatability (**Supplementary Figure 3**).

**FIGURE 5:**
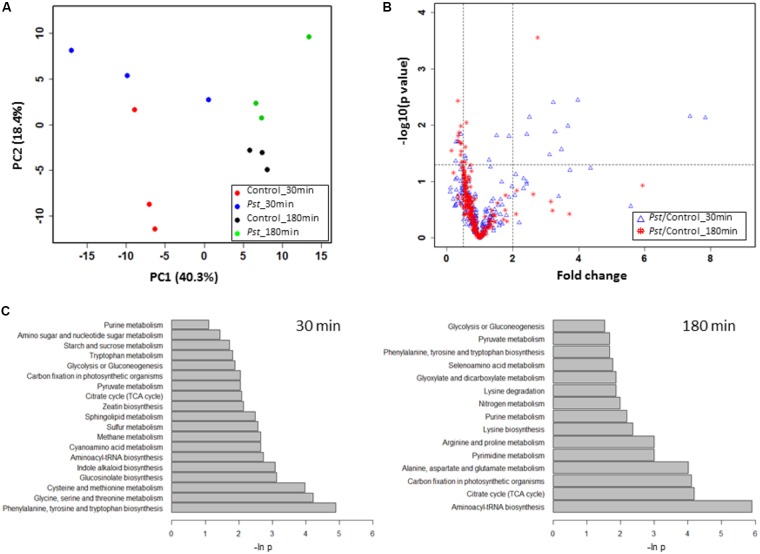
*Pst* DC3000 modulation of plant metabolism. **(A)** PCA analysis of plant metabolites under *Pst* DC3000 infection. An unsupervised PCA was performed with the MRM data from four experimental groups. The first two principle components were plotted, and the percentages of variance explained by each of them were labeled on the respective axes. **(B)** Significantly changed metabolites under *Pst* DC3000 infection. The horizontal dash line denoted –log10 of the significance cutoff (*p* = 0.05), and the two vertical dashed lines denoted 0.5 and 2 as the cut off thresholds for fold change, respectively. **(C)** Pathway analysis of plant metabolites in response to *Pst* DC3000 infection. Enriched pathways were shown at 30 min (left) and 180 min after incubation (right).

The normalized data in **Supplementary Table 3** were subjected to an unsupervised PCA analysis, in which the first two components explained 58.7% of the total variance in the global metabolite profiles (**Figure 5A**). The clear separation between the control and the *Pst* DC3000 treated samples demonstrated that the guard cells reprogramed the cellular metabolic pathways. A full list of the metabolites identified and their levels can be found in **Supplementary Table 3**. Notably, samples with different bacterial incubation times can also be separated based on their metabolite profiles.

### Signaling and Primary Metabolite Changes in Plant Response to Pathogen Infection

To identify plant metabolites showing changes in response to pathogen infection, two criteria were applied to the quantitative MRM data: (i) at least twofold change (FC) between the *Pst* DC3000 treated and control samples at different time points, and (ii) statistical significance at the level of *p* < 0.05. Here we chose two time points, i.e., 30 min when stomata were closing and 180 min when stomata are open. The volcano plot in **Figure 5B** showed that majority of the metabolites did not show significant changes as they clustered at FC of 1. At 30 min after *Pst* DC3000 infection, 16 metabolites were significantly altered (**Table 1**). Notably, 11 of them increased in levels and most of them are involved in primary metabolism. In addition, signaling compounds such as cyclic adenosine diphosphate ribose (cADPR) and cyclic guanosine monophosphate (cGMP) were also significantly increased. In contrast, only one of the 13 significantly changed metabolites at 180 min increased after the pathogen treatment. The other 12 metabolites decreased, and they include primary metabolites such as ATP and amino acids (**Table 1**), highlighting the importance of primary metabolites in plant defense. The significantly changed metabolites were subjected to pathway analysis. As shown in **Figure 5**, at 30 min the top three most influenced pathways were related to amino acid metabolism. At 180 min, the top three drastically changed pathways consist of aminoacyl-tRNA biosynthesis, citrate cycle, and carbon fixation. Interestingly, the biosynthesis pathways of phenylalanine, tyrosine, and tryptophan were also affected at 180 min.

**Table 1:** Significantly changed metabolites in Arabidopsis epidermal peels (EPs) upon *Pst* DC3000 infection.

30 min			180 min		
Compound	FC	*p*-value	Compound	FC	*p*-value
2-Deoxyguanosine-5-mono phosphate	7.8	7E-03	Caffeic acid	2.8	3E-04
Adenosine-3-monophosphate	7.4	7E-03	Guanine	0.5	5E-02
Uridine-5-monophosphate	4.0	4E-03	Phosphoenolpyruvic acid	0.5	4E-02
Cyclic guanosine monophosphate	3.7	1E-02	Lysine	0.4	1E-02
Tricine	3.5	3E-02	Glutamine	0.4	3E-02
2-Deoxyadenosine-5-MonoPhosphate	3.3	1E-02	Arginine	0.4	2E-02
ATP	3.2	4E-03	Dehydroascorbic acid	0.4	1E-02
Fructose	3.1	3E-02	*cis*-aconitic acid	0.4	2E-02
Luteolin-7-beta-glucoside	2.5	7E-03	Uridine	0.4	3E-02
Methylthiobutyric acid	2.4	1E-02	Sarcosine	0.3	2E-02
Serine	0.4	5E-02	Alanine	0.3	2E-02
Phosphoenolpyruvic acid	0.4	1E-02	*N*-alpha -L ornithine	0.3	4E-03
Tryptophan	0.3	2E-02	Traumatic acid	0.1	3E-02
Salicin	0.3	4E-02			
Traumatic acid	0.3	2E-02			
Cyclic adenosine diphosphate ribose	Inf^∗^	2E-02			

*^∗^The compound was only detected in the treated samples*.

## Discussion

Application of stable isotope labeling to studying metabolomics of plant-microbe interactions has not been reported before. The method developed here allows for differentiation of plant metabolome from the metabolome of the infecting pathogens. The workflow is simple and robust, requiring isotope labeling of *Pst* DC3000 in an isotope medium and simple washing steps after the incubation of bacteria with plant tissues or cells. The medium is commercially available and affordable, and the washing steps are fast and efficient. For assays in which separating the pathogen from plant cells by washing is difficult or impossible, isotopic labeling of the microbial metabolome alone should be adequate to differentiate the two different metabolomes. Clearly, this method has the potential to discover and quantify important plant metabolites that otherwise would be skewed or masked due to interference of the microbial metabolites. Importantly, it has broad applications in studying metabolic interactions between microbes and any organisms.

Recently, significant progress has been made to understand the intricate plant defense networks that involve the generation of reactive oxygen species (Camejo et al., 2016), activation of protein kinases (Zhang et al., 2017), induction of pathogenesis-related (*PR*) gene expression, synthesis of phytoalexin (Piasecka et al., 2015) and activation of programmed cell death (Teh and Hofius, 2014). However, the contribution of primary metabolites to plant immune response is not well-understood. The role of primary metabolism was traditionally thought to provide energy for defense. Recent studies pointed a regulatory role of primary metabolites during plant–pathogen interaction (Rojas et al., 2014; Asai et al., 2017; Murcia et al., 2017). For instance, carbohydrates such as glucose, fructose and sucrose were shown to upregulate *PR* gene expression (Xiao et al., 2000; Bolouri Moghaddam and Van den Ende, 2012). In addition, genes in photorespiration and amino acid metabolism were found to be responsive to pathogen infections (Schafer et al., 2004; Less et al., 2011). In line with these studies, we found that carbohydrates, ATP and amino acids showed significant changes in the treated samples compared to the controls. For example, fructose showed more than a threefold increase at 30 min, and most amino acids showed a decrease at 180 min after pathogen infection. This suggested that carbohydrate metabolism (along with purine metabolism) responded rapidly to pathogen infection, while changes at the amino acid levels occurred later. Time-course studies with small intervals and more data points would allow a better understanding of the metabolite flux in response to *Pst* DC3000. Interestingly, serine showed a decrease at 30 min, indicating a possible low photorespiration activity since the precursor of serine, phosphoglycolate is produced during photorespiration (Montero et al., 2016). Alternatively, serine could be incorporated into proteins or degraded.

During early stages of the guard cell immune response, a number of signaling metabolites showed increases in abundance. For example, cADPR abundance was high at 30 min compared to control. In animal cells, cADPR is a secondary messenger that mobilizes Ca^2+^ to modulate a diverse array of cellular processes (Lee, 2012). A regulatory role for cADPR in plant cells has been studied in abscisic acid (ABA)-mediated stress responses (Wu et al., 1997). ABA is a phytohormone that regulates guard cell signaling to modulate stomatal movement, and it is also implicated in plant immune signal transduction (Zhang et al., 2014; Mittler and Blumwald, 2015; Balmant et al., 2016; Lievens et al., 2017). Another signaling molecule that showed increased abundance was cGMP, which is also an intracellular secondary messenger. It has been demonstrated that cGMP regulates numerous events in plant growth and development (Dubovskaya et al., 2015), and it functions downstream of ROS in ABA-induced stomatal closure (Dubovskaya et al., 2011). Thus, it is possible that cADPR and cGMP could serve as converging points between ABA signaling and pathogen-triggered immunity signaling.

At 180 min after infection, most of the significant changes involved decreases in amino acids (alanine, lysine, glutamine, and arginine) and nucleosides (guanine and uridine) in plants. Ward et al reported that both alanine and glutamine showed slightly increased abundance in Arabidopsis infected by *Pst* DC3000 at 8 h infection (Ward et al., 2010). The discrepancy could be due to the different time points when the observations were made as plants adjust metabolic pathways dynamically. It would be interesting to determine whether the decrease is caused by decreased biosynthesis and/or increased degradation. Another possible explanation is that EPs with enriched guard cells, instead of the whole leaves, were used in this study. Distinct metabolic patterns between guard cells and mesophyll cells have been demonstrated (Jin et al., 2013; Misra et al., 2015). Accordingly, using EPs with stomatal guard cells could reveal metabolic changes specific in guard cells that otherwise would be missed. Nonetheless, both studies demonstrated that the regulation of amino acid accumulation may play a role in plant immune response. Intriguingly, glutamate, instead of glutamine identified in this study, was reported to mediate stomatal closure in both Arabidopsis and fava bean (Yoshida et al., 2016). In contrast, the function of nucleosides is elusive during plant defense responses. It has been reported that nucleotide sugars are differentially changed in *TRANSPARENT TESTA8* (*TT8*) mutant plants, which are less tolerant to biotic stress (Rai et al., 2016). However, the mechanisms underlying the role of nucleoside and their derivatives need further investigation.

Both targeted and untargeted metabolomics approaches had been utilized in studying plant pathogen interactions (Aliferis et al., 2014; Warth et al., 2015; Cajka and Fiehn, 2016). While a targeted approach such as MRM in this study enables accurate quantification, it lacks the capacity to discover new metabolites (Menni et al., 2017). In order to screen for unknown compounds that are essential in plant pathogen interactions, an untargeted metabolomics approach serves as an attractive alternative. Regardless of the platform, labeling the pathogen metabolites with heavy isotopes would allow profiling of plant metabolites with no complications introduced by the pathogen. On the other hand, it also opens the door to characterize metabolites and metabolic pathways unique in the pathogen cells, which play a critical role in pathogenesis. It should be noted that this isotope-labeling approach is not without caveats. Bacterial cells may incorporate plant-derived metabolites for nutrition if extended incubation time is allowed. In addition, bacterial cell division during the infection would contribute to heavy isotope dilution. Using water for the incubation in this study can prevent bacteria from reproducing quickly on the peels. Therefore, the current protocol may be best suited to study the early stage of plant–pathogen interaction.

## Conclusion

The isotope-labeling of microbes reported here addresses a major problem in current metabolomics of plant–pathogen interactions. Profiling of plant-specific metabolites was performed by LC-MRM-MS following exposure of Arabidopsis EPs with stomatal guard cells to isotope-labeled *Pst* DC3000. The overall metabolic patterns in the control and treated samples were distinct. The time-resolved analysis also revealed significantly changed plant metabolites in response to the bacterial infection, which include signaling and primary metabolites. The discovery of these metabolites provides important clues to further studies toward a better understanding of plant–pathogen interaction at the metabolomic level. Furthermore, the method developed here can be applied to other interaction systems where species-specific metabolite dynamics needs to be characterized.

## Author Contributions

QP conducted the isotope labeling, collected the plant materials, and performed the metabolomics experiments. TZ established the bacterial washing method, assisted in experimental design, and conducted the data analysis and paper drafting. YW participated in statistical analysis. WK assisted in the plant material collection and mass spectrometry experiments. XY participated in the experimental design and supervised personnel. SC designed the experiments, oversaw the work, and finalized the manuscript. All the authors read the manuscript and provided comments.

## Conflict of Interest Statement

The authors declare that the research was conducted in the absence of any commercial or financial relationships that could be construed as a potential conflict of interest.
