# Supplementary figures and images for: Metabolomics of Early Stage Plant Cell–Microbe Interaction Using Stable Isotope Labeling
Source: Front Plant Sci. 2018 Jun 5;9:760. doi: 10.3389/fpls.2018.00760 (PMC5996122; doi:10.3389/fpls.2018.00760)

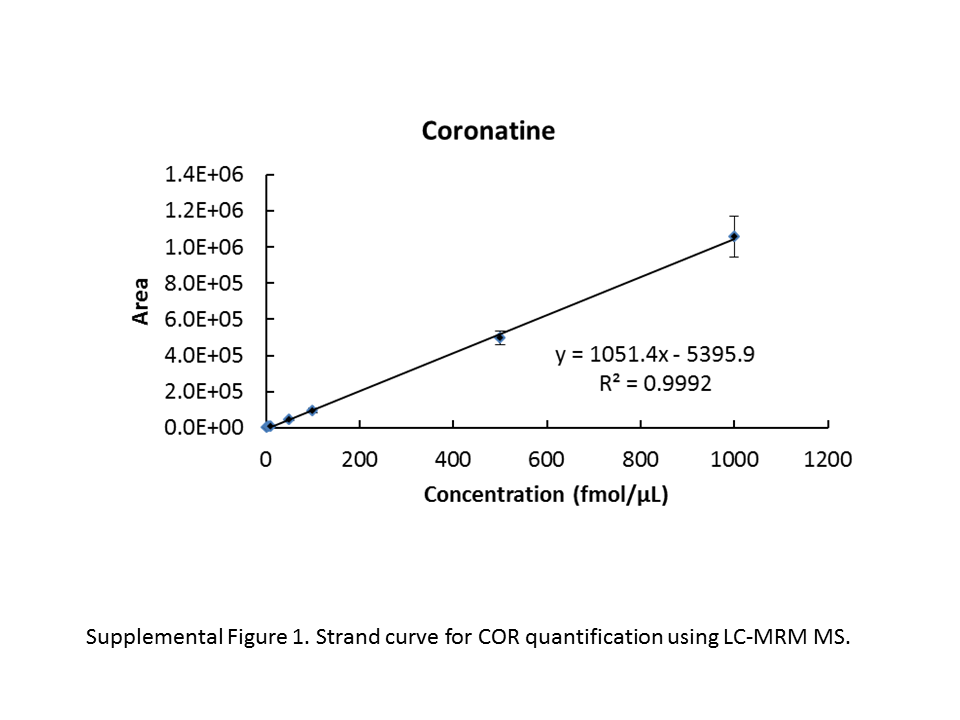

Supplement: Supplementary file 1 [file Image_1.TIF]

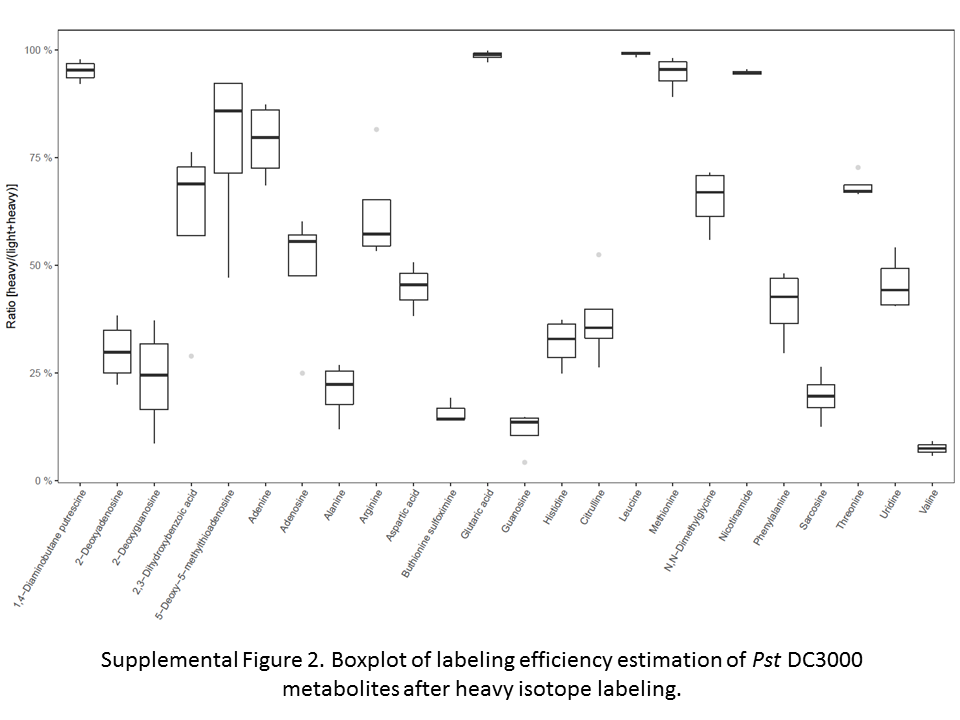

Supplement: Supplementary file 2 [file Image_2.TIF]

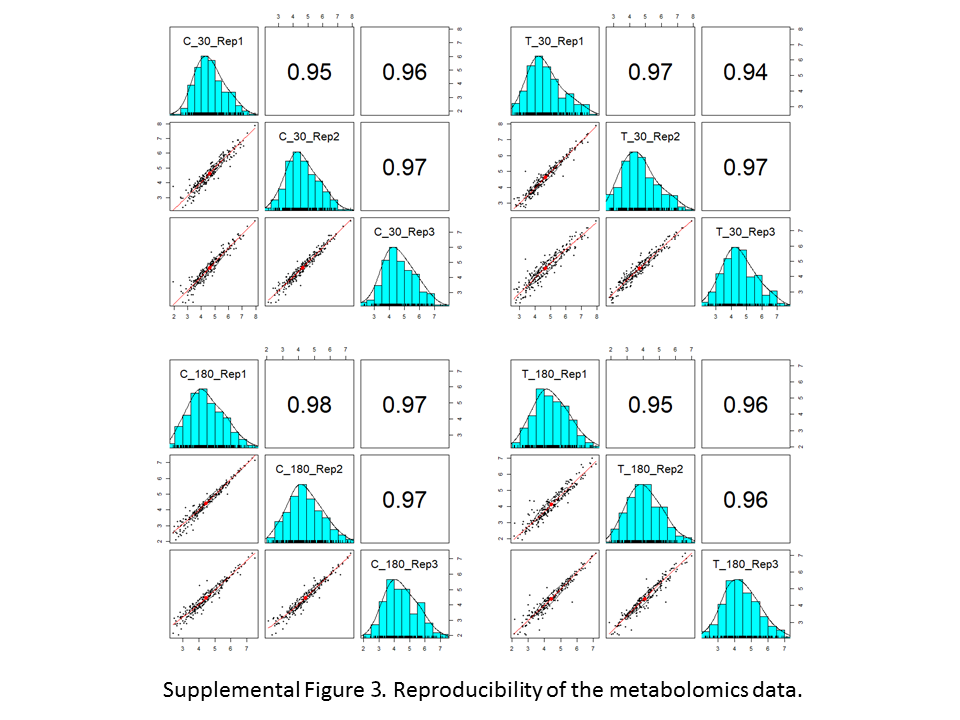

Supplement: Supplementary file 3 [file Image_3.TIF]
