# Supplementary material for: Metabolomics of Early Stage Plant Cell–Microbe Interaction Using Stable Isotope Labeling
Source: Front Plant Sci. 2018 Jun 5;9:760. doi: 10.3389/fpls.2018.00760 (PMC5996122; doi:10.3389/fpls.2018.00760)
Supplement: Supplementary file 5 [file Table_2.pdf]

Supplemental Table 2. MRM transitions for light/heavy metabolites pairs.

No.	Name	Formula	MW	Q1	Q3	Structure	Fragmentation
**Negative mode**							
1	Malonic acid	$C_3H_4O_4$	104.06	103	59.2	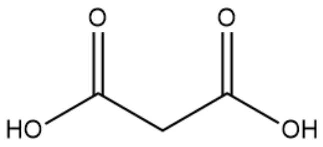	Chemical Formula: $C_2H_3O_2^{\bullet}$<br>Exact Mass: 59.01<br>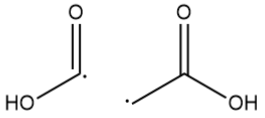<br>Chemical Formula: $CHO_2^{\bullet}$<br>Exact Mass: 45.00
				106	61.2		
2	GTP (Guanosine 5'-triphosphate sodium salt hydrate)	$C_{10}H_{16}N_5O_{14}P_3 \cdot xNa^+$	523.18	522.3	158.9	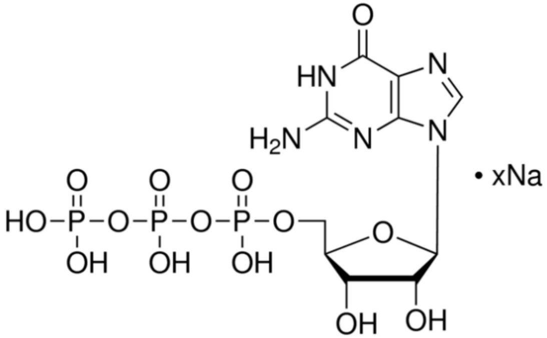	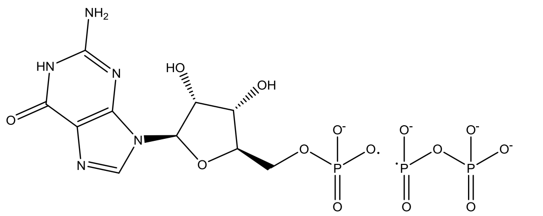<br>Chemical Formula: $C_{10}H_{12}N_5O_8P^{\bullet-}$<br>Exact Mass: 361.04<br>Chemical Formula: $O_6P_2^{\bullet 3-}$<br>Exact Mass: 157.92
				537.3	158.9		
3	Glucose	$C_6H_{12}O_6$	180.16	179.0	59.1	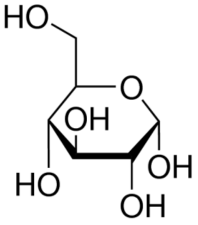	Chemical Formula: $C_2H_3O_2^{\bullet}$<br>Exact Mass: 59.01<br>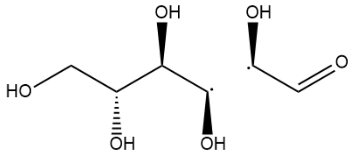<br>Chemical Formula: $C_4H_9O_4^{\bullet}$<br>Exact Mass: 121.05
				185.0	61.1		
4	Fumaric acid	$C_4H_4O_4$	116.07	115.1	70.9	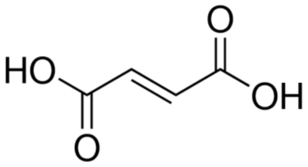	Chemical Formula: $C_3H_3O_2^{\bullet}$<br>Exact Mass: 71.01<br>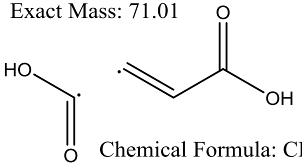<br>Chemical Formula: $CHO_2^{\bullet}$<br>Exact Mass: 45.00
				119.1	73.9		

5	Ethylmalonic acid	$C_5H_8O_4$	132.11	131.1	87.1	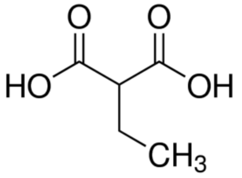	Chemical Formula: $CHO_2^{\bullet}$<br>Exact Mass: 45.00<br>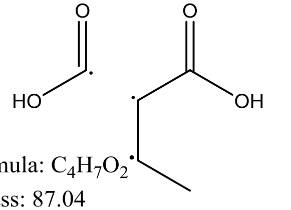<br>Chemical Formula: $C_4H_7O_2^{\bullet}$<br>Exact Mass: 87.04
				136.1	91.1		
6	L-Dihydroorotic acid	$C_5H_6N_2O_4$	158.11	157.1	112.9	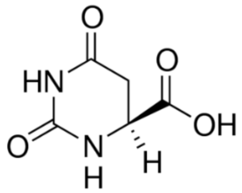	Chemical Formula: $C_4H_5N_2O_2^{\bullet}$<br>Exact Mass: 113.04<br>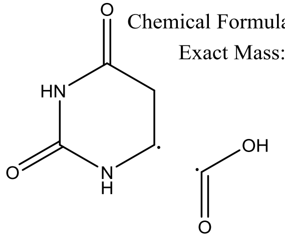<br>Chemical Formula: $CHO_2^{\bullet}$<br>Exact Mass: 45.00
				164.1	118.9		
7	Pimelic acid	$C_7H_{12}O_4$	160.17	159.1	96.8	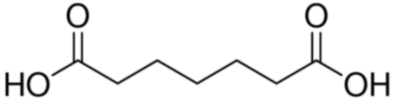	Chemical Formula: $CHO_2^{\bullet}$<br>Exact Mass: 45.00<br>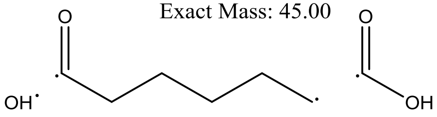<br>Chemical Formula: $C_6H_{10}O^{2\bullet}$<br>Exact Mass: 98.07
				166.1	102.8		
8	Xanthosine	$C_{10}H_{12}N_4O_6$	284.23	283	150.9	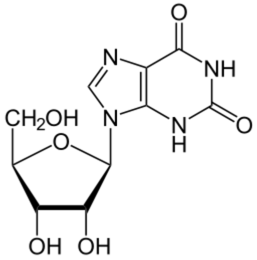	Chemical Formula: $C_5H_3N_4O_2^{\bullet}$<br>Exact Mass: 151.03<br>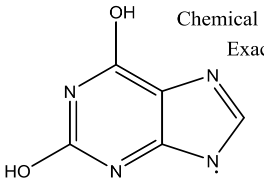<br>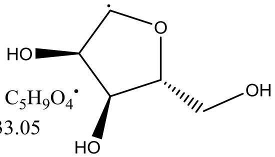<br>Chemical Formula: $C_5H_9O_4^{\bullet}$<br>Exact Mass: 133.05
				297	159.9		

**Positive mode**							
9	1,4-Diaminobutane	$C_4H_{12}N_2$	88.15	89.1	72	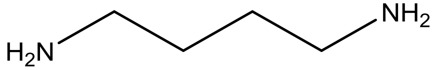	Chemical Formula: $C_4H_{10}N^{\bullet}$<br>Exact Mass: 72.08<br>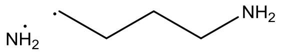<br>Chemical Formula: $H_2N^{\bullet}$<br>Exact Mass: 16.02
				95.1	77		
10	Sarcosine	$C_3H_7NO_2$	89.09	89.9	44.1	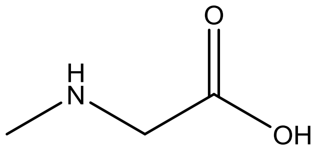	Chemical Formula: $CHO_2^{\bullet}$<br>Exact Mass: 45.00<br>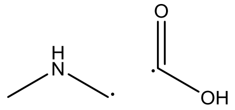<br>Chemical Formula: $C_2H_6N^{\bullet}$<br>Exact Mass: 44.05
				93.9	47.1		
11	Alanine	$C_3H_7NO_2$	89.09	90	44	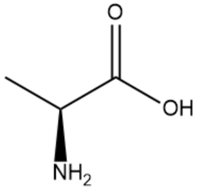	Chemical Formula: $CHO_2^{\bullet}$<br>Exact Mass: 45.00<br>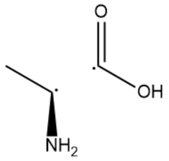<br>Chemical Formula: $C_2H_6N^{\bullet}$<br>Exact Mass: 44.05
				94	47		
12	N,N-Dimethylglycine	$C_4H_9NO_2$	103.12	104	58.1	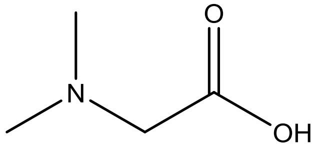	Chemical Formula: $CHO_2^{\bullet}$<br>Exact Mass: 45.00<br>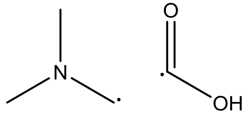<br>Chemical Formula: $C_3H_8N^{\bullet}$<br>Exact Mass: 58.07
				109	62.1		

13	Threonine	**$C_4H_9NO_3$**	119.12	120	74	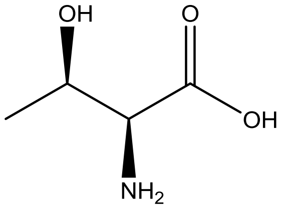	Chemical Formula: $CHO_2^{\bullet}$<br>Exact Mass: 45.00<br>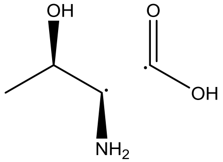<br>Chemical Formula: $C_3H_8NO^{\bullet}$<br>Exact Mass: 74.06
				125	78		
14	Leucine	$C_6H_{13}NO_2$	131.17	132	86	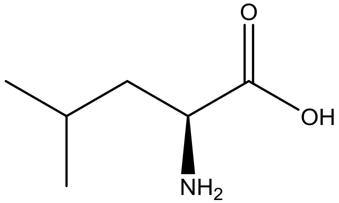	Chemical Formula: $CHO_2^{\bullet}$<br>Exact Mass: 45.00<br>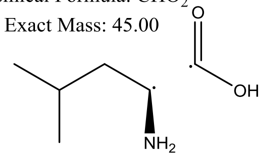<br>Chemical Formula: $C_5H_{12}N^{\bullet}$<br>Exact Mass: 86.10
				139	92		
15	Glutaric acid	$C_5H_8O_4$	132.12	133.1	87	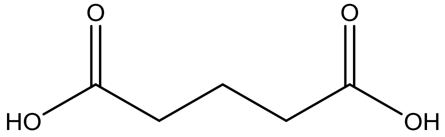	Chemical Formula: $C_4H_7O_2^{\bullet}$<br>Exact Mass: 87.04<br>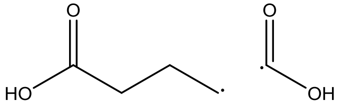<br>Chemical Formula: $CHO_2^{\bullet}$<br>Exact Mass: 45.00
				138.1	91		
16	Histidine	$C_6H_9N_3O_2$	155.15	156	110	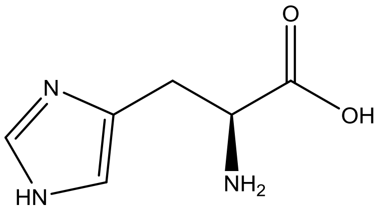	Chemical Formula: $CHO_2^{\bullet}$<br>Exact Mass: 45.00<br>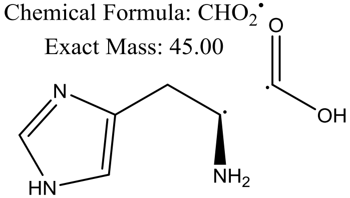<br>Chemical Formula: $C_5H_8N_3^{\bullet}$<br>Exact Mass: 110.07
				165	118		

17	Arginine	$C_6H_{14}N_4O_2$	174.20	175	70	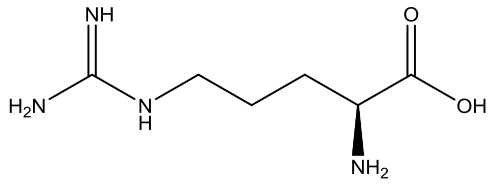	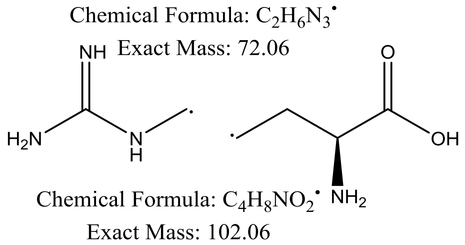
				185	75		
18	L-Citrulline	$C_6H_{13}N_3O_3$	175.19	176.1	70	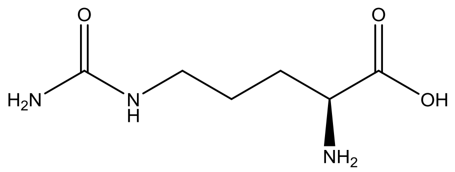	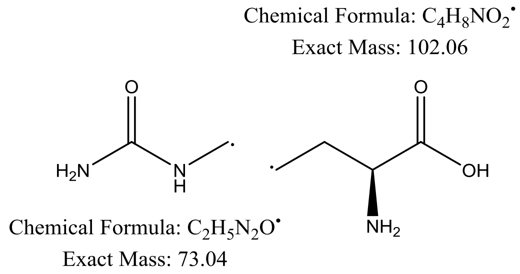
				185.1	74		
19	D-Mannitol	$C_6H_{14}O_6$	182.17	183.1	147.1	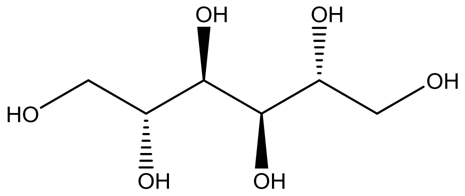	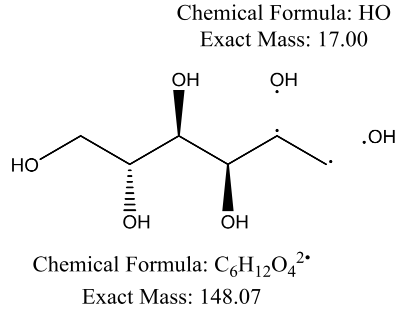
				189.1	153.1		
20	Nicotinamide	$C_6H_6N_2O$	122.13	123.1	79.8	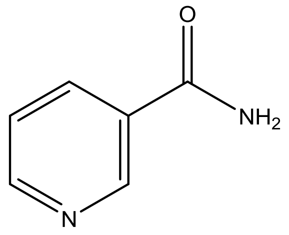	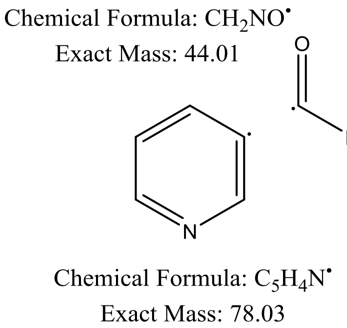
				131.1	85.8		

21	Aspartic Acid	$C_4H_7NO_4$	133.10	134	74	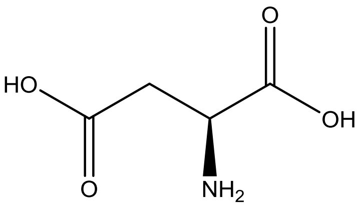	Chemical Formula: $C_2H_4NO_2^{\bullet}$<br>Exact Mass: 74.02<br>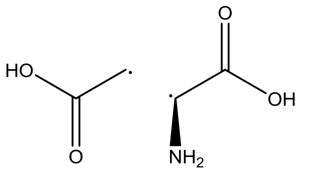<br>Chemical Formula: $C_2H_3O_2^{\bullet}$<br>Exact Mass: 59.01
				139	77		
22	Adenine	$C_5H_5N_5$	135.13	135.88	119	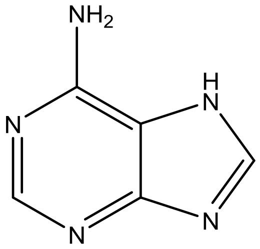	Chemical Formula: $H_2N^{\bullet}$<br>Exact Mass: 16.02<br>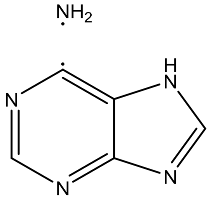<br>Chemical Formula: $C_5H_3N_4^{\bullet}$<br>Exact Mass: 119.04
				145.88	128		
23	Methionine	$C_5H_{11}NO_2S$	149.05	150	104	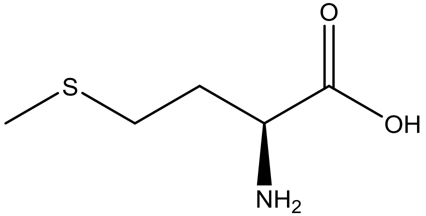	Chemical Formula: $CHO_2^{\bullet}$<br>Exact Mass: 45.00<br>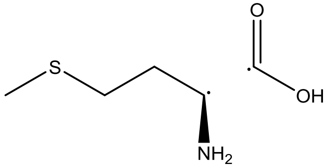<br>Chemical Formula: $C_4H_{10}NS^{\bullet}$<br>Exact Mass: 104.05
				156	109		
24	Valine	$C_5H_{11}NO_2$	117.15	118	71.9	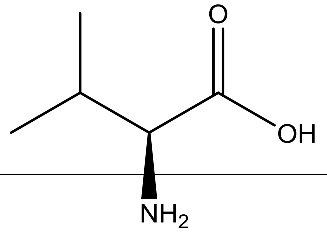	Chemical Formula: $CHO_2^{\bullet}$<br>Exact Mass: 45.00<br>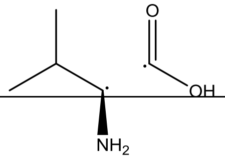<br>Chemical Formula: $C_4H_{10}N^{\bullet}$<br>Exact Mass: 72.08

				124	76.9		
25	Pyroglutamic acid	$C_5H_7NO_3$	129.12	129.8	84	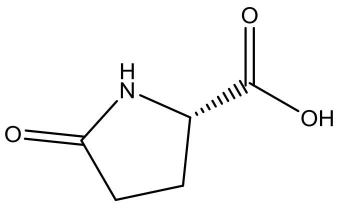	Chemical Formula: $CHO_2^{\bullet}$<br>Exact Mass: 45.00<br>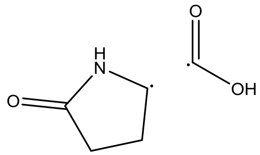<br>Chemical Formula: $C_4H_6NO^{\bullet}$<br>Exact Mass: 84.04
				135.8	89		
26	2,3-Dihydroxybenzoic Acid	$C_7H_6O_4$	154.12	155.04	137.04	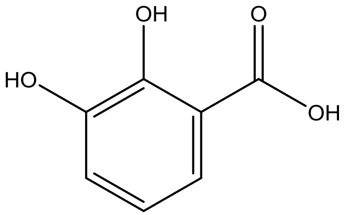	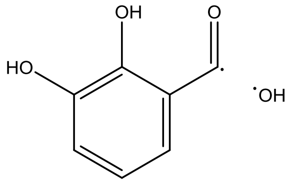<br>Chemical Formula: $HO^{\bullet}$<br>Exact Mass: 17.00<br>Chemical Formula: $C_7H_5O_3^{\bullet}$<br>Exact Mass: 137.02
				162.04	144.04		
27	Phenylalanine	$C_9H_{11}NO_2$	165.19	166	120	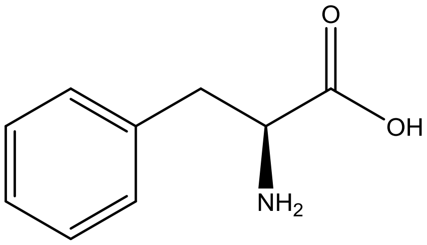	Chemical Formula: $CHO_2^{\bullet}$<br>Exact Mass: 45.00<br>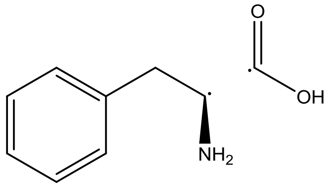<br>Chemical Formula: $C_8H_{10}N^{\bullet}$<br>Exact Mass: 120.08
				176	129		
28	Uridine	$C_9H_{12}N_2O_6$	244.2	245.069	113	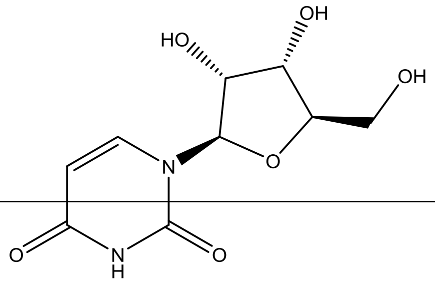	Chemical Formula: $C_5H_9O_4^{\bullet}$<br>Exact Mass: 133.05<br>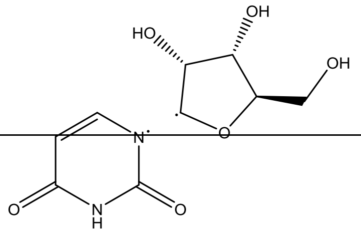<br>Chemical Formula: $C_4H_3N_2O_2^{\bullet}$<br>Exact Mass: 111.02

				256.069	119		
29	2-Deoxyadenosine	$C_{10}H_{13}N_5O_3$	251.25	252.1<br>267.1	136.1<br>146.1	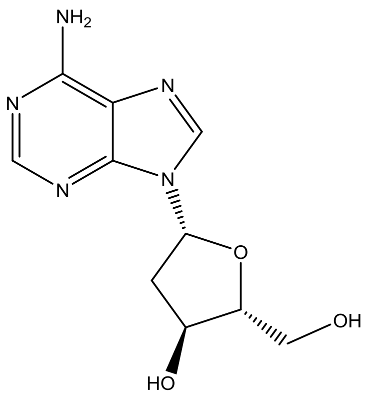	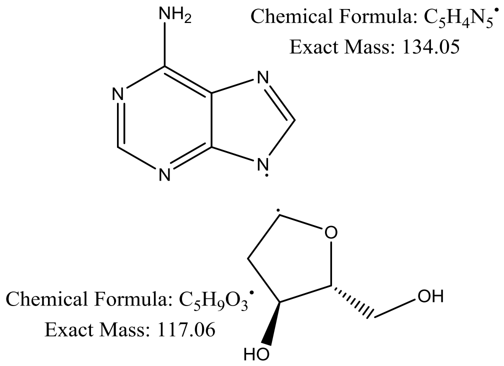
30	2-Deoxyguanosine monohydrate	$C_{10}H_{13}N_5O_4$	267.25	268.2<br>283.2	152.1<br>162.1	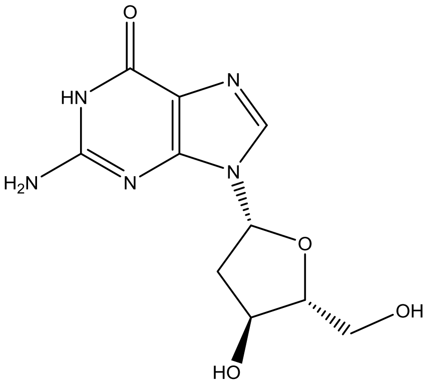	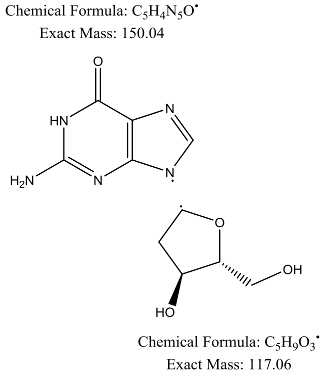
31	Adenosine	$C_{10}H_{13}N_5O_4$	267.25	268.24	136	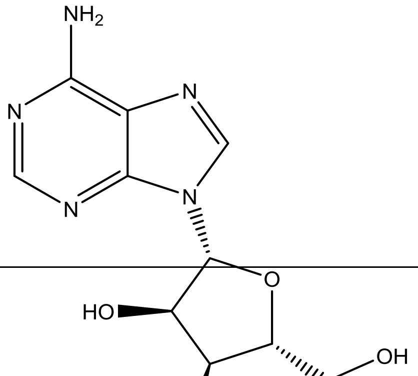	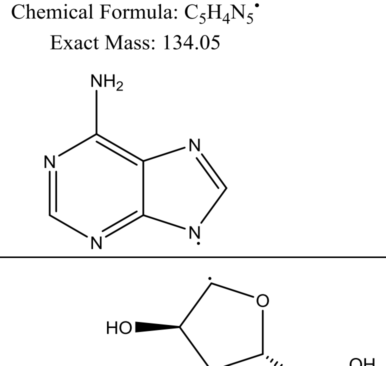

				283.24	146		
32	Guanosine	$C_{10}H_{13}N_5O_5$	283.24	284	152	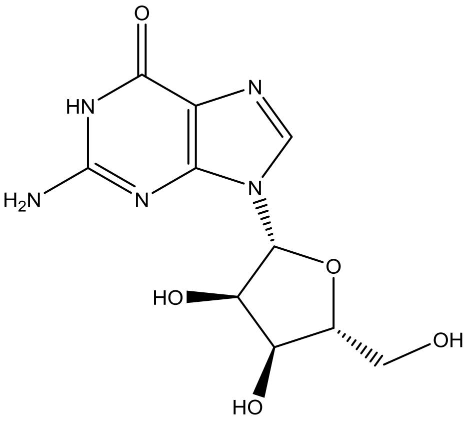	Chemical Formula: $C_5H_4N_5O^{\bullet}$<br>Exact Mass: 150.04<br>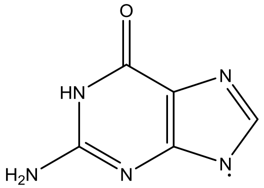<br>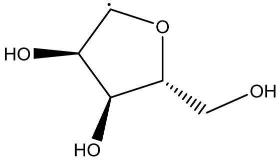<br>Chemical Formula: $C_5H_9O_4^{\bullet}$<br>Exact Mass: 133.05
				299	162		
33	5-Deoxy-5-methylthio-adenosine	$C_{11}H_{15}N_5O_3S$	297.33	298.1	136.1	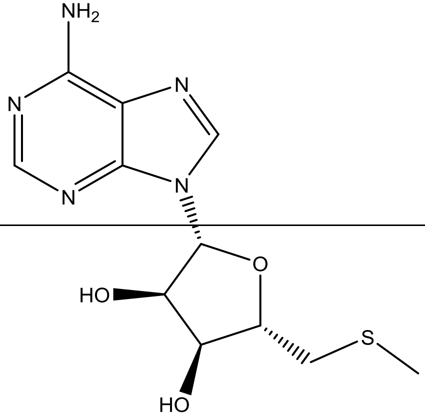	Chemical Formula: $C_5H_4N_5^{\bullet}$<br>Exact Mass: 134.05<br>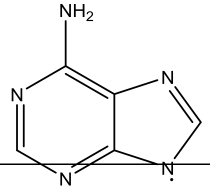<br>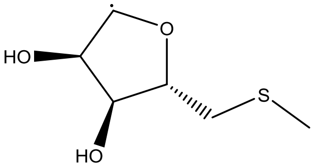<br>Chemical Formula: $C_6H_{11}O_3S^{\bullet}$<br>Exact Mass: 163.04

				314.1	146.1		
34	Allantoin	$C_4H_6N_4O_3$	158.12	159.1<br>167.1	116.2<br>122.2	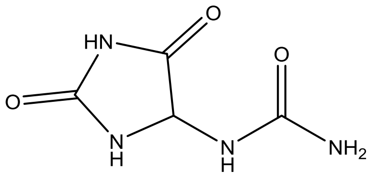	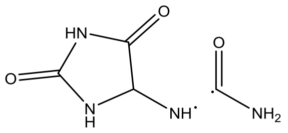<br>Chemical Formula: $C_3H_4N_3O_2^{\bullet}$<br>Exact Mass: 114.03<br>Chemical Formula: $CH_2NO^{\bullet}$<br>Exact Mass: 44.01
35	AMP	$C_{10}H_{14}N_5O_7P$	347.22	348.4<br>363.4	135.9<br>145.9	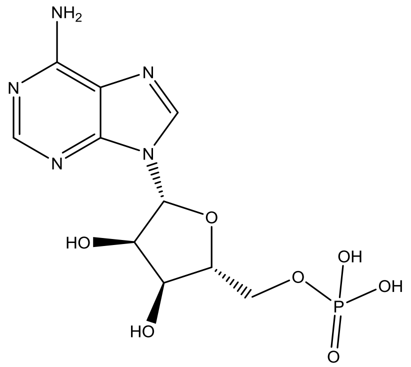	Chemical Formula: $C_5H_4N_5^{\bullet}$<br>Exact Mass: 134.05<br>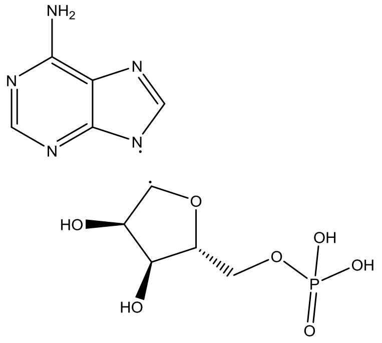<br>Chemical Formula: $C_5H_{10}O_7P^{\bullet}$<br>Exact Mass: 213.02
36	Proline	**$C_5H_9NO_2$**	115.13	116<br>122	70<br>75	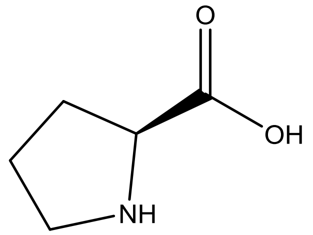	Chemical Formula: $CHO_2^{\bullet}$<br>Exact Mass: 45.00<br>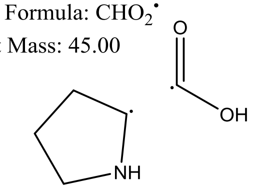<br>Chemical Formula: $C_4H_8N^{\bullet}$<br>Exact Mass: 70.07

37	Serine	**$C_3H_7NO_3$**	105.09	105.9	59.8	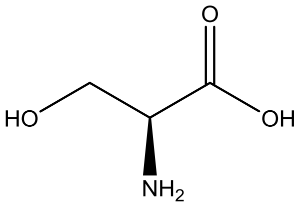	Chemical Formula: $CHO_2^{\bullet}$<br>Exact Mass: 45.00<br>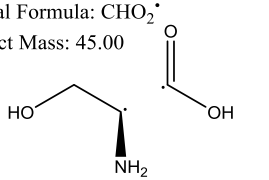<br>Chemical Formula: $C_2H_6NO^{\bullet}$<br>Exact Mass: 60.04
				109.9	62.8		
38	Asparagine	**$C_4H_8N_2O_3$**	132.12	133	74	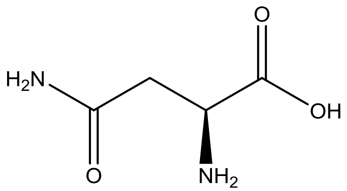	Chemical Formula: $C_2H_4NO_2^{\bullet}$<br>Exact Mass: 74.02<br>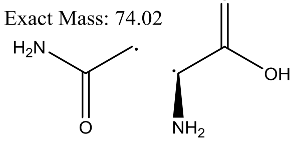<br>Chemical Formula: $C_2H_4NO^{\bullet}$<br>Exact Mass: 58.03
				139	77		
39	Sinapinic acid	$C_{11}H_{12}O_5$	224.21	225.2	206.9	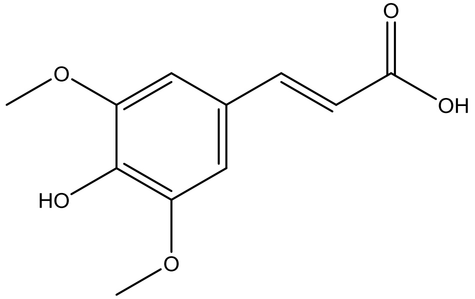	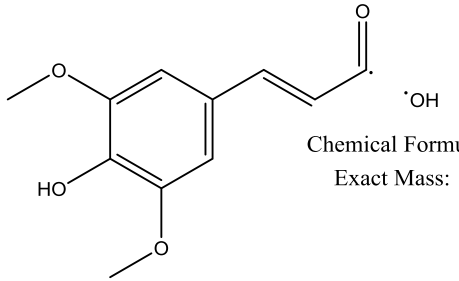<br>Chemical Formula: $HO^{\bullet}$<br>Exact Mass: 17.00<br>Chemical Formula: $C_{11}H_{11}O_4^{\bullet}$<br>Exact Mass: 207.07
				236.2	217.9		
